# JAK-STAT and AKT pathway-coupled genes in erythroid progenitor cells through ontogeny

**DOI:** 10.1186/1479-5876-10-116

**Published:** 2012-06-07

**Authors:** Vladan P Cokic, Bhaskar Bhattacharya, Bojana B Beleslin-Cokic, Constance T Noguchi, Raj K Puri, Alan N Schechter

**Affiliations:** 1 Laboratory of Experimental Hematology, Institute for Medical Research, University of Belgrade, Belgrade, 11129, Serbia; 2 Tumor Vaccines and Biotechnology Branch, Division of Cellular and Gene Therapies, Center for Biologics Evaluation and Research, Food and Drug Administration, Bethesda, MD, 20892, USA; 3 Institute of Endocrinology, Diabetes and Diseases of Metabolism, School of medicine, University Clinical Center, Belgrade, Serbia; 4 Molecular Medicine Branch, National Institute of Diabetes and Digestive and Kidney Diseases, National Institutes of Health, Bethesda, MD, 20892, USA

**Keywords:** Erythroid progenitors, Microarray, Ontogeny, JAK-STAT pathway, AKT pathway

## Abstract

**Background:**

It has been reported that the phosphatidylinositol 3-kinase (PI3K)-AKT signaling pathway regulates erythropoietin (EPO)-induced survival, proliferation, and maturation of early erythroid progenitors. Erythroid cell proliferation and survival have also been related to activation of the JAK-STAT pathway. The goal of this study was to observe the function of EPO activation of JAK-STAT and PI3K/AKT pathways in the development of erythroid progenitors from hematopoietic CD34^+^ progenitor cells, as well as to distinguish early EPO target genes in human erythroid progenitors during ontogeny.

**Methods:**

Hematopoietic CD34^+^ progenitor cells, isolated from fetal and adult hematopoietic tissues, were differentiated into erythroid progenitor cells. We have used microarray analysis to examine JAK-STAT and PI3K/AKT related genes, as well as broad gene expression modulation in these human erythroid progenitor cells.

**Results:**

In microarray studies, a total of 1755 genes were expressed in fetal liver, 3844 in cord blood, 1770 in adult bone marrow, and 1325 genes in peripheral blood-derived erythroid progenitor cells. The erythroid progenitor cells shared 1011 common genes. Using the Ingenuity Pathways Analysis software, we evaluated the network pathways of genes linked to hematological system development, cellular growth and proliferation. The *KITLG, EPO, GATA1, PIM1* and *STAT3* genes represent the major connection points in the hematological system development linked genes. Some JAK-STAT signaling pathway-linked genes were steadily upregulated throughout ontogeny (*PIM1, SOCS2, MYC, PTPN11*), while others were downregulated (*PTPN6, PIAS, SPRED2*). In addition, some JAK-STAT pathway related genes are differentially expressed only in some stages of ontogeny (*STATs, GRB2, CREBB*). Beside the continuously upregulated (*AKT1, PPP2CA, CHUK, NFKB1*) and downregulated (*FOXO1, PDPK1, PIK3CG*) genes in the PI3K-AKT signaling pathway, we also observed intermittently regulated gene expression (*NFKBIA, YWHAH*).

**Conclusions:**

This broad overview of gene expression in erythropoiesis revealed transcription factors differentially expressed in some stages of ontogenesis. Finally, our results show that EPO-mediated proliferation and survival of erythroid progenitors occurs mainly through modulation of JAK-STAT pathway associated *STATs, GRB2* and *PIK3* genes, as well as AKT pathway-coupled *NFKBIA* and *YWHAH* genes.

## Background

The regulation of erythropoiesis is a very complex process requiring the coordination of different signaling pathways and molecular reactions. Many transcription factors controlling globin gene expression, such as GATA binding proteins 1/2 (GATA1/2), Krüppel-like factor (KLF1), nuclear factor erythroid-derived 2 (NFE2), have been identified and characterized. The erythroid-specific transcription factor GATA1 is a direct activator of the beta (β)-globin gene [1]. GATA1 homodimerizes and interacts with other transcription factors, such as erythroid KLF1 and friend of GATA1 (FOG), further contributing to activation of delta (δ)-, gamma (γ)-, and β-globin promoters [2]. KLF1 is a zinc finger transcription factor that activates the β-globin gene promoter [3]. The protein FOG is co-expressed with GATA1 during embryonic development in erythroid cells [4]. DRED was identified as a repressor of the epsilon (ϵ)-globin gene, it appears to prevent binding of KLF1 to the ϵ-globin gene promoter and silences ϵ-globin expression during definitive erythropoiesis [5]. Another erythroid-specific transcription factor, called Kruppel-like factor 11 (KLF11), activates also γ- and ϵ-globin genes in vitro [6]. In primitive erythropoiesis GATA2 is primarily expressed, but later in ontogeny GATA1 expression predominates [7]. Downregulation of GATA2 is important for progression of erythroid cell differentiation [8]. A nuclear protein, special AT-rich binding protein 1 (SATB1), regulates genes through targeting chromatin remodeling and increases ϵ-globin and decreases γ-globin gene expression [9]. Activation of globin production by transcription factor NFE2 is stimulated by cAMP-dependent protein kinase (PKA) in erythroid cells [10].

Genome extensive profiling has been used in several studies of erythroid differentiation [11-13]. Some of these were primarily concerned about different stages of erythropoiesis to recognize potential transcription factors regulating gene expression during terminal erythropoiesis [14]. Increased apoptotic activity has been found for peripheral blood (PB)-derived hematopoietic progenitor CD34^+^ cells compared to bone marrow (BM)-derived CD34^+^ cells [15]. It has been reported that erythropoietin (EPO) and stem cell factor (SCF) mediated synergistic expansion of primary erythroid precursors activating JAK-STAT, phosphatidylinositol 3-kinase (PI3K) and MAPK pathways [16]. Activation of each of the JAK-STAT, MAPK p42/44 or PI3K-AKT pathways alone is not sufficient either to stimulate cell proliferation or inhibit apoptosis of human CD34^+^ cells and erythroblasts. Erythroid proliferation appears more related to simultaneous activation of JAK-STAT and MAPK p42/44 whereas the effect on cell survival correlates better with activation of PI3K-AKT, JAK-STAT and MAPK p42/44 pathways. EPO and SCF inhibit apoptosis of early erythroid CD34^+^ burst-forming units (BFU-E) progenitors and erythroblasts [17]. In addition, the EPO activation of JAK-STAT pathway has an important role in inhibiting apoptosis of human hematopoietic cells, similarly to the PI3K-AKT axis [18].

It has been reported that feeder layers of stroma from human fetal liver (FL), cord blood (CB), and adult bone marrow (BM) enhance CD34^+^ hematopoietic progenitor cell proliferation and erythropoiesis [19]. We have examined the growth and erythroid differentiation capacity of CD34^+^ cells only, already committed toward hematopoiesis in fetal and adult hematopoietic cells. Using this approach, we evaluated a broad range of genes expressed in erythroid progenitors derived from hematopoietic cells through ontogeny. The broad comparison of erythropoiesis-related signaling pathways highlighted statistically significant changes in gene expression among the examined cells. Regarding its significance, we choose to focus on JAK-STAT and PI3K-AKT signaling pathways during erythroid differentiation. The most prominent genes are v-myc (myelocytomatosis viral oncogene homolog (*MYC*)) and pim-1 oncogene (*PIM1*) in the JAK-STAT pathway, whereas in the PI3K-AKT pathway emerge the heat shock protein 90 kDa alpha (*HSP90AA1*) and protein phosphatase 2 alpha (*PPP2CA*) genes. After activation of v-akt (murine thymoma viral oncogene homolog 1 (*AKT1*)), cell survival is promoted via nuclear factor of kappa light polypeptide gene enhancer in B-cells 1 (*NFKB1*) and conserved helix-loop-helix ubiquitous kinase (*CHUK*) according to its elevated gene expression in the PI3K-AKT pathway. Activation of JAK-STAT pathway coupled genes is observed via STATs and protein tyrosine phosphatase, non-receptor type 11 (*PTPN11*) gene expression linked to transcription regulation and differentiation. We describe upregulated and downregulated genes during erythroid differentiation of hematopoietic progenitor cells, with the emphasis on JAK-STAT and PI3K-AKT coupled genes, to define the mechanism of erythropoiesis.

## Methods

### Liquid erythroid cell cultures

To follow erythropoiesis through ontogeny, we isolate hematopoietic CD34^+^ progenitor cells from corresponding fetal and adult hematopoietic tissues and stimulate in vitro erythroid differentiation. Adult PB mononuclear cells are isolated from buffy coats of three healthy donors (NIH Blood bank) using Lymphocyte Separation Medium (BioWhittaker, Walkersville, MD) and CD34^+^ cells are purified by positive immunomagnetic selection with MACS cell isolation system (Miltenyi Biotec, Auburn, CA). Commercial FL-derived CD34^+^ cells (Cambrex Bio Science, Inc., Walkersville, MD), CB- and BM-derived CD34^+^ cells (AllCells LLC, Berkeley, CA) are also isolated by positive immunomagnetic selection (Miltenyi Biotec). To stimulate erythroid differentiation, the labeled CD34^+^ cells of all ontogenic stages are cultured in the medium that contains 30% fetal bovine serum (FBS), 2 mmol/L glutamine, 100 U/ml penicillin, 100 μg/ml streptomycin, 10% deionized bovine serum albumin, 10 mmol/L mercaptoethanol, 1 mmol/L dexamethasone, 33 μg/ml holo-transferrin, 10 ng/ml SCF, 1 ng/ml IL-3 and 1 ng/ml GM-CSF (Sigma, St. Louis, MO), and 1 U/ml human recombinant EPO (Amgen Inc, Thousand Oaks, CA) [20]. For microarray analysis, erythroid progenitors are isolated at day 6 of erythroid cell culture at 37°C and 5% CO_2_ with balanced 95% room air. Anti-CD71 Tricolor is used for cell staining (Beckman-Coulter, Miami, FL). Cells are fixed in PBS containing 4% formaldehyde, and acquire on an LSRII flow cytometer (BD Biosciences, San Jose, CA). Data are analyzed with Flowjo software (Tree Star, San Carlos, CA).

### Isolation of total RNA

We use the RNeasy protocol for isolation of total RNA from erythroid progenitor cells (Qiagen, Valencia, CA) according to the manufacturer's instructions. Concentration and integrity of total RNA is assessed using an 8453 UV/Visible Spectrophotometer (Hewlett-Packard GmbH, Waldbronn, Germany) and Agilent 2100 Bioanalyzer Software (Agilent Technologies, Waldbronn, Germany).

### Microarray studies

In microarray studies, the numbers of total genes overexpressed in erythroid cells of CB, BM and PB origin are determined from three independent samples as biological repeats. On the other hand in case of FL-derived samples, the number of total overexpressed genes is determined in independent duplicate samples. High quality oligonucleotide glass arrays are produced containing a total of 16,659 seventy-mer oligonucleotides chosen from 750 bases of the 3′ end of each ORF (Operon Inc. Valencia, CA). The arrays are produced in house by spotting oligonucleotides on poly-L-lysine coated glass slides by Gene Machines robotics (Omnigrid, San Carlos, CA). We have followed the MIAME (minimum information about a microarray experiment) guidelines for the presentation of our data [21].

#### Probe preparation

Total human universal RNA (HuURNA) isolated from a collection of adult human tissues to represent a broad range of expressed genes from both male and female donors (BD Biosciences, Palo Alto, CA) serve as a universal reference control in the competitive hybridization. All examined samples are hybridized against HuURNA. The correlation coefficients among those biological repeats themselves are consistently ≥ 0.8, which documented the quality of hybridization and consistency of expression among the replicates of all examined erythroid progenitors. Labeled cDNA probes are produced as described [22]. Briefly, 5 μg of total RNA is incubated at 70°C for 5 minutes along with 1 μl of aminoallyl-oligo dT primer and quickly chilled for 3 minutes. Then, 2 μl 10X first strand buffer, 1.5 μl SSII enzyme (Stratagene, La Jolla, CA), 1.5 μl 20X aminoallyl dUTP and 2 μl of 0.1 M DTT are added and incubated for 90 minutes at 42°C. After incubation, volume of the reaction mixture is raised to 60 μl with 40 μl of DEPC water. cDNA is purified by the MinElute column (Qiagen). 300 μl of Binding buffer PB is added to the coupled cDNA, and the mixture applied to the MinElute column, and centrifuged for 1 minute at 10.000 rpm. After discharging the flow-through, 600 μl of washing buffer PE is added to the column, and centrifuged for 1 minute at 10.000 rpm. The flow-through is discharged and the washing repeated. Then the columns are placed into a fresh eppendorf tube and 15 μl elution buffer added to the membrane, incubated for 1 minute at room temperature, centrifuged for 1 minute at 10.000 rpm and probe collected. The probe is dried in speed-vac for 16 minutes. Finally, 5 μl of 2X coupling buffer and 5 μl Cy3 and Cy5 dye (GE Healthcare Bio-Sciences Corp., Piscataway, NJ) are mixed into the control (HuURNA) and experimental cDNAs (huES cell-derived) respectively and incubated at room temperature in dark for 90 minutes. After incubation, the volume is raised to 60 μl by 50 μl DEPC water and then cDNA is purified by the MinElute column and eluted with 13 μl elution buffer by centrifugation.

#### Hybridization

For hybridization, 36 μl hybridization mixture [26 μl cDNA mixture, 1 μl (10 μg) COT-1 DNA, 1 μl (8–10 μg) poly(dA), 1 μl yeast total RNA (4 μg), 6 μl 20X SSC and 1 μl 10% SDS] is pre-heated at 100°C for 2 minutes and cooled for 1 minute. Total volume of probe is added on the array and covered with cover slip. Slides are placed in hybridization chamber and 20 μl water is added to the slide, and incubated overnight at 65°C. Slides are then washed for 2 minutes each in 2X SSC, 1X SSC and 0.1X SSC and spin-dried.

#### Data filtration, normalization, and analysis

Microarray slides are scanned in both Cy3 (532 nm) and Cy5 (635 nm) channels using Axon GenePix 4000B scanner (Axon Instruments, Inc., Foster City, CA) with a 10-micron resolution. Scanned microarray images are exported as TIFF files to GenePix Pro 3.0 software for image analysis. The raw images are collected at 16-bit/pixel resolutions with 0 to 65,535 count dynamic range. The area surrounding each spot image is used to calculate a local background and subtracted from each spot before the Cy5:Cy3 ratio calculation. The average of the total Cy3 and Cy5 signal gives a ratio that is used to normalize the signals. Each microarray experiment is globally normalized to make the median value of the log2-ratio equal to zero. The Loess normalization process corrects for dye bias, photo multiplier tube voltage imbalance, and variations between channels in the amounts of the labeled cDNA probes hybridized. The data files representing the differentially expressed genes are then created. For advanced data analysis, gpr and jpeg files are imported into microarray database, and normalized by software tools provided by NIH Center for Information Technology (http://nciarray.nci.nih.gov/). Spots with confidence interval of 99 (≥ 2 fold) with at least 150-fluorescence intensity for both channel and 30 μm spot size are considered as good quality spots for analysis. We gathered a set of 8,719 erythroid cells gene expression data derived from 11 datasets that have been posted on the National Center for Biotechnology Information (NCBI) Gene Expression Omnibus (GEO) database.

### Statistical analysis

The one way ANOVA with Tukey's Multiple Comparison tests and paired t test are applied using Prism 4 software (GraphPad Software Inc., San Diego, CA) for measurement of statistical significance in microarray analysis during ontogenesis. Ingenuity Pathways Analysis is a software application that enables identification of the biological mechanisms, pathways and functions most relevant to the genes of interest [http://www.ingenuity.com].

## Results

### Gene expression patterns in erythroid progenitor cells during ontogeny

In the presence of EPO and other cytokines, CD34^+^ hematopoietic progenitor cells are differentiated in vitro into erythroid progenitor cells. We already reported the steady increase in adult hemoglobin and decline in fetal hemoglobin levels during *in vitro* erythroid differentiation of PB CD34^+^ cells [20]. At day 6 of erythroid cell culture, the erythroid progenitor cells of examined ontological stages are labeled as 100% CD71^+^ (a well-known early marker of erythroid differentiation) are isolated for microarray analysis. Moreover, we perform flow cytometry of three additional differentiation markers CD34, CD36 and glycophorin A and despite variation in their expression we did not find the statistical significance at day 6 of erythroid culture (not shown). The total number of expressed genes per ontogenic stage, evaluated by microarray analysis, is presented in Table 1 (first column). The microarray data discussed in this publication we deposited in NCBI’s GEO database and are accessible through GEO Series accession number GSE37869 (http://www.ncbi.nlm.nih.gov/geo/query/acc.cgi?acc=GSE37869). Presence of individual genes in two-thirds of examined samples per cell group (66% filtering) reduced largely the total gene expression (second column, Table 1). The total gene expression is more than doubled in erythroid progenitor cells of CB-derived cells in comparison to other cells. Adult PB-derived erythroid progenitor cells express the least quantity of genes. A total of 43 genes were highly expressed (two-fold and higher) in FL, 84 genes in CB, 90 in BM, and 29 genes in PB-derived erythroid progenitor cells (Table 1). Overexpression of 1011 genes is common in all cells during ontogeny (after 66% filtering). In addition to globins, the common highly expressed genes in erythroid progenitor cells are proteoglycan 2 (*PRG2*), Charcot-Leyden crystal protein (*CLC*), serglycin (*SRGN*), eosinophil peroxidase (*EPX*) and *MYC*. To examine effect of FBS on erythropoiesis, we decide to follow erythropoietic markers γ and β globins in the same in vitro erythroid culture conditions with or without FBS. However, the reduction of γ globin gene expression and γ/β ratio did not reach statistical significance in FBS-deprived culture conditions (not shown).

**Table 1:** Number of total genes in examined cells and quantification of overexpressed genes versus HuURNA

**Filtering-derived cells**	**No total**	**total**	**66%**		
			**≥ 1.5 fold**	**≥ 2 fold**	**≥ 3 fold**
**FL**	**5900**	**1755**	**150**	**43**	**10**
**CB**	**6667**	**3844**	**325**	**84**	**14**
**BM**	**7002**	**1770**	**247**	**90**	**13**
**PB**	**3794**	**1325**	**83**	**29**	**2**

*FL*-fetal liver, *CB*-cord blood, *BM*-bone marrow, *PB*-peripheral blood.

### Microarray analysis of gene expression profiles in erythroid progenitor cells

**Figure 1:**
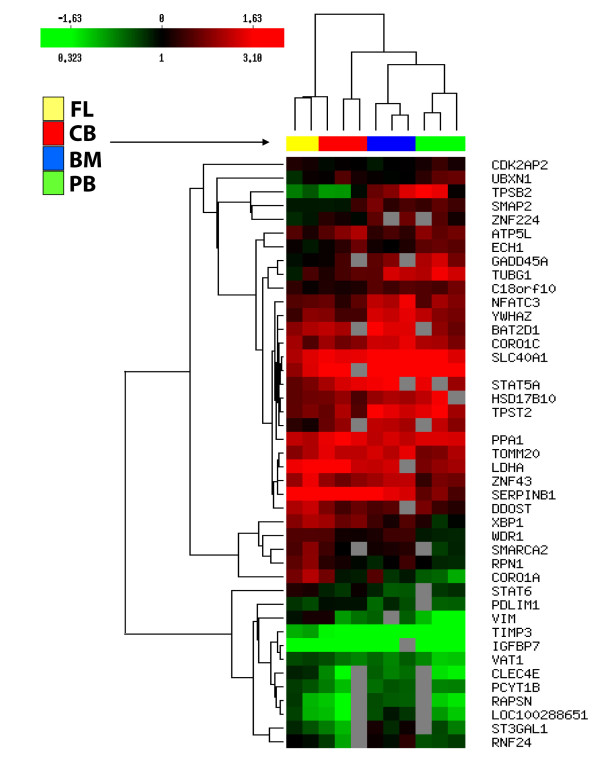
** Hierarchical clustering of genes expressed during ontogeny.** Hierarchical clustering of statistically significant genes determined by ANOVA and expressed during ontogeny (left side). The color indicates the relative fold expression of each gene, with red indicating higher expression, green indicating negative expression, black representing not changed expression, while gray stands for absent expression per each examined sample. The total gene expression of erythroid progenitor cells from various cells is also clustered (above image) representing similarities among various cells.

To distinguish genes with statistically significant expression in erythroid progenitors through ontogeny we perform comparisons using the t-test (Table 2-4). During microarray analysis genes are upregulated or downregulated compared to HuURNA, used as a control alongside each sample. We present upregulated genes compared to HuURNA determined by t-test in Table 2. The other statistically significant genes that have the same pattern of expression, as genes in Table 2, are presented in Additional file 1. The *ERAF* gene related to erythroid differentiation and hematological system development is upregulated in BM tissue (Table 2). The same patterns of expression and ratio as *ERAF* gene are also observed for the following genes: *ENY2, GSTO1, HMGB2, HPS4, HSD17B10, METTL13* (Additional file 1). We also separate downregulated genes compared to HuURNA determined by t-test (Table 3, Additional file 2). The same pattern of expression, statistical significance and ratio as *GNB1* gene, elevated in CB-derived cells, is also observed for the *CYB5R3, ILF3* and *NKX2-5* genes. The same patterns of expression and ratio as *PDGFRA* gene, upregulated in BM tissue, are also observed for the *ERGIC1* and *RAPSN* genes (Additional file 2). Some genes are differentially upregulated and downregulated in various stages, so we present them as special group of diverse genes in statistical analysis by t-test (Table 4, Additional file 3). *STAT5A* and *STAT5B* have the highest gene expression in BM-derived erythroid cells (Tables 2). The same patterns of expression, statistical significance and ratio as *STAT5B* gene are observed for the following genes: *HIGD1A, KHSRP, LYSMD3, SF1, SPEN, ACOT9, TACC3, VDAC2*. Besides statistical analysis performed by t-test between two group of cells, we also make ANOVA analysis of the common genes among all four ontogenic stages and present them in Additional file 4. This extended statistical analysis also reveal that *HDAC1* and *SERPINB1* genes are significantly increased in FL-derived erythroid progenitor cells (Tables 2). *HDAC2* gene expression is largely increased throughout ontogeny with the highest level also in FL-derived cells (not shown). Apoptotic *IGFBP7* gene is downregulated, mostly in adult derived cells (Table 3). In addition, these ANOVA determined genes are also shown in hierarchical clustering analysis, as well as clustering of individual samples of all examined cells (Figure 1).

**Table 2:** Statistically significant genes by t-test up-regulated vs. HuURNA among examined cells. p < 0.01 (≤, ≥), p < 0.05 (<, >)

**Gene Name**	**Description vs.**	**F**	**F**	**F**	**C**	**C**	**B**
		**C**	**B**	**P**	**B**	**P**	**P**
**ABCE1**	ATP-binding cassette, sub-family E memb. 1		<				
ACLY	ATP citrate lyase				>		
AP1B1	adaptor-related protein complex1,β1 subunit	>				<	
ATP1B3	ATPase, Na^+^/K^+^ transporting, β3 polypeptide			>			
**BAT2D1**	BAT2 domain containing 1						>
**BTF3L3**	basic transcription factor 3-like 3	<				>	
**CAPZA2**	capping protein (actin fil.) muscle Z-line, α2				≤		
CLK2	CDC-like kinase 2						>
CORO1C	coronin, actin binding protein, 1 C			>	<		>
CSDE1	cold shock domain containing E1, RNA-bind	<					
DDOST	dolichyl-diphosphooligos-prot glycosyltransf	>	>	>			
ECH1	enoyl Coenzyme A hydratase 1 peroxisomal			≤			≤
ENO1	enolase 1, (alpha)					>	
**ERAF**	erythroid associated factor		<				
FADS2	fatty acid desaturase 2	<					
GOLIM4	golgi integral membrane protein 4				≤		
HDAC1	histone deacetylase 1	>					
HSPA5	heat shock 70 kDa protein 5				>		≥
HSPA8	heat shock 70 kDa protein 8			>			
**LDHA**	lactate dehydrogenase A			>		>	>
**MYBL2**	v-myb myeloblastosis viral oncog hom-like 2				<		
NDUFAB1	NADH dehydrog ubiquinone 1 α/β subcomp1			<			<
NFATC3	nucl fact of activ T-cel cytopl calcineur-dep 3		<		<		
**PDIA6**	protein disulfide isomerase family A m6			>			
PPA1	pyrophosphatase (inorganic) 1			<			
PSMB3	proteasome (prosome) subunit, β type, 3		>				
RPL18A	ORF		>				<
RPN1	ribophorin I	>		≥			
**SERPINB1**	serpin peptidase inhibitor, clade B m1			≥		≥	
**STAT5A**	Signal transd. and activator of transcript. 5A	<	<				
TFDP3	transcription factor Dp family, member 3	≤		<			
TIMM23	transl of inner mitoch memb 23 hom ngemp		<				
**TPST2**	tyrosylprotein sulfotransferase 2		<				
TUBG1	tubulin, gamma 1			<		≤	
TXNL1	thioredoxin-like 1			<			
YWHAZ	tyrosine 3-/tryptophan 5-monooxygenase zeta pp		<		≤		>
ZNF224	zinc finger protein 224		≤		<		
ZNF43	zinc finger protein 43				<		>

nuclear gene encoding mitochondrial protein (ngemp), member (m). Bolded genes – expression >1.5 fold vs. HuURNA; *F*-fetal liver, *C*-cord blood, *B*-bone marrow, *P*-peripheral blood.

**Table 3:** Statistically significant genes by t-test down-regulated vs. HuURNA among examined cells. p < 0.01 (≤, ≥), p < 0.05 (<, >)

**Gene Name**	**Description vs.**	**F**	**F**	**F**	**C**	**C**	**B**
		**C**	**B**	**P**	**B**	**P**	**P**
ADRB3	adrenergic, β-3-, receptor					<	
ATP6C	ATPase, H + transporting, lysosomal 16kD	<				>	
BTBD10	BTB (POZ) domain containing 10		≤				
BTRC	beta-transducin repeat containing						>
CABP2	Calcium binding protein 2						>
CLEC4E	C-type lectin domain family 4, member E		≥	≥			>
COQ10B	coenzyme Q10 homolog B (S. cerevisiae)				<		≥
DVL3	dishevelled, dsh homolog 3				<		
F2R	coagulation factor II (thrombin) receptor					>	
FPR1	N-formylpeptide receptor fMLP-R98 ORF			>			
GSTM1	glutathione S-transferase mu 1			>			
**IGFBP7**	insulin-like growth factor binding protein 7			≥		>	>
MINK1	Misshapen-like kinase 1 (zebrafish)			<			
**MT1A**	metallothionein 1A	<					
NPIPL3	nuclear pore complex interact. protein-like 3			<			
PDGFRA	platelet-derived growth factor receptor, α				<		>
PDLIM1	PDZ and LIM domain 1	<			≥	>	
PHLDA1	pleckstrin homology-like domain family A m1					>	
PSMC4	proteasome (prosome) 26 S sub, ATPase, 4				>		
SP2	Sp2 transcription factor		>	>			
SQSTM1	sequestosome 1			<		<	
ST3GAL1	ST3 β-galactoside α-2,3-sialyltransferase 1				<		
**TIMP3**	TIMP metallopeptidase inhibitor 3	>	>	>		>	
UBE2D3	ubiquitin-conjugating enzyme E2D 3		<				
UBXN1	UBX domain protein 1						<
VAT1	vesicle amine transport protein 1 homolog			>			>

nuclear gene encoding mitochondrial protein (ngemp), member (m). Bolded genes – expression >1.5 fold vs. HuURNA. *F*-fetal liver, *C*-cord blood, *B*-bone marrow, *P*-peripheral blood.

**Table 4:** **Statistically significant by t-test genes down-/up-regulated vs. HuURNA among examined cells. p < 0.01 (**≤, ≥**), p < 0.05 (**<, >**)**

**Gene Name**	**Description vs.**	**F**	**F**	**F**	**C**	**C**	**B**
		**C**	**B**	**P**	**B**	**P**	**P**
ATP6V1B2	ATPase, H + transport, lysosomal 56/58 kDa, V1 sub B2			≥			>
BRP44	brain protein 44	<		≤		≤	
CD24	CD24 molecule		>	>			
CDK2AP2	cyclin-dependent kinase 2 associated prot. 2	>				<	<
CLTA	clathrin, light chain (Lca)			≤			
COMT	catechol-O-methyltransferase		≥		≥		
**CORO1A**	coronin, actin binding protein, 1A			≥			
GADD45A	growth arrest and DNA-damage-inducible α		<	<			
ITGB2	integrin, beta 2						>
KIFC1	HSET mRNA for kinesin-related protein				<		
LSR	lipolysis stimulated lipoprotein receptor			≥			
PSMB6	proteasome (prosome) subunit, β type, 6			<			
SMAP2	small ArfGAP2		<	<			
SMARCA2	SWI/SNF related, matrix associated, actin depend regul of chromat, subfam a, memb 2			>			>
STAT6	signal transd & activat of transcr 6, IL-4 ind		>	≥			
THYN1	thymocyte nuclear protein 1		<				
TMX2	thioredoxin-related transmembrane protein 2		<				
TPSB2	tryptase beta 2		<		<	<	
VASP	vasodilator-stimulated phosphoprotein			≥			
**VIM**	vimentin		>	>			
WDR1	WD repeat domain 1			≥		>	≥
XBP1	X-box binding protein 1		>	≥	>	≥	

nuclear gene encoding mitochondrial protein (ngemp), member (m). Bolded genes – expression >1.5 fold vs. HuURNA. F-fetal liver, C-cord blood, B-bone marrow, P-peripheral blood.

### Pathways linked to hematological system development

**Figure 2:**
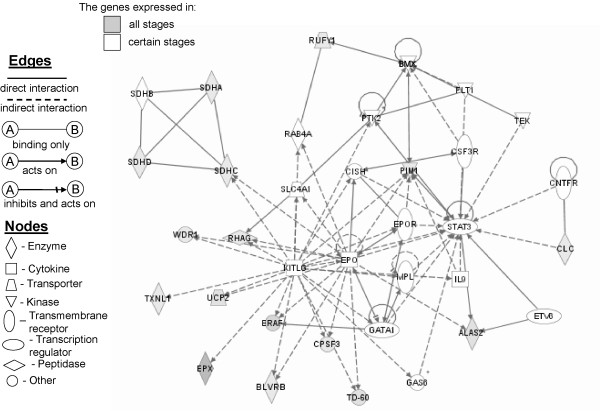
** Hematological system development, cellular growth and proliferation.** Using Ingenuity Pathways Analysis software we created the network pathway of genes related to hematological system development. White nodes represent expressed genes in some stages, while gray nodes represent genes expressed in all stages throughout ontogeny. The intensity of gray color is in positive correlation with a level of gene expression in erythroid progenitors.

Using the Ingenuity Pathways Analysis software, we evaluate the network pathway of genes linked to hematological system development (Figure 2). As shown in Figure 2, KITLG (SCF), EPO, GATA1 and STAT3 represent the major junction points in hematological system development. The effects of KITLG are only indirect toward other related molecules, whereas STAT3 is the major target point for direct or indirect effects of linked molecules (Figure 2). In our microarray analysis, *KITLG* and *EPO* gene expression are downregulated in BM- and PB-, whereas *STAT3* gene expression is slightly upregulated in erythroid progenitor cells and more notably in CB- and BM-derived cells. *GATA1* gene expression is considerably upregulated in all cells during ontogeny, except a low level in FL-derived cells. KITLG and EPO linked *ERAF* and *UCP2* gene expression are increased during ontogeny, but reached maximum in adult derived cells (Table 2, Additional file 1). The expression of genes related to hematological system development throughout ontogeny (Figure 2) have the following characteristics: *WDR1* gene expression, induced by EPO and KITLG, is decreased only in PB-derived cells, with the top level in erythroid progenitor cells of FL origin (Table 4). *EPX* gene expression is significantly upregulated in FL- and CB-derived erythroid cells compared to PB-derived cells. Rh-associated glycoprotein (*RHAG*) has decreased expression in BM-, whereas cytokine inducible SH2-containing protein (*CISH*) and *PIM1* have increased expression in BM- and PB-derived erythroid cells. CLC, colony stimulating factor 3 receptor (*CSF3R*), cleavage and polyadenylation specific factor 3 (*CPSF3*) and small GTP binding protein (*RAB4A*) genes have increased expression, whereas thioredoxin-like 1 (*TXNL1*) has decreased expression in FL-derived erythroid progenitor cells. EPO receptor (*EPOR*) gene expression is increased in CB- and BM-derived erythroid cells. Regarding succinate dehydrogenase complex gene expression, subunit A (*SDHA*) is also increased in FL-, subunit B (*SDHB*) in CB- and BM-, subunit D (*SDHD*) in PB-, while subunit C (*SDHC*) is decreased in BM-derived erythroid cells.

### Proliferation and survival of erythroid cells

**Figure 3:**
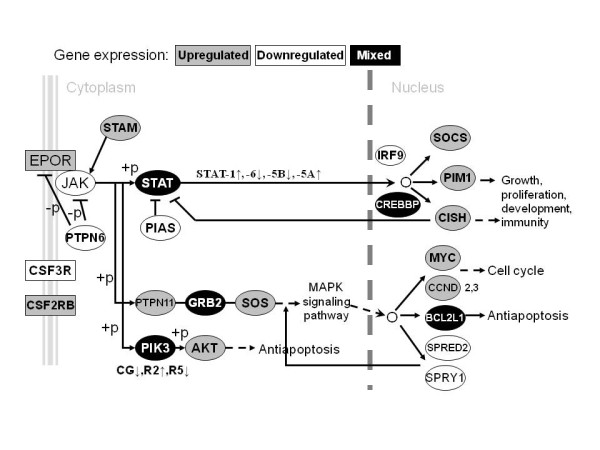
** JAK-STAT signaling pathway.** The expression of genes linked to JAK-STAT signaling pathway through ontogeny. (+p) phosphorylation, (−p) dephosphorylation, → stimulation, ⟂ inhibition, → translocation, ↓ decreased gene expression, ↑ increased gene expression.

EPO stimulates the JAK-STAT pathway during erythroid differentiation [16]. We also use EPO for erythroid differentiation of CD34^+^ hematopoietic progenitor cells in liquid cultures. We present JAK-STAT signaling pathway related gene expression in Figure 3, as determined by microarray analysis. Some JAK-STAT pathway related genes are persistently upregulated throughout ontogeny (*PIM1, SOCS2, MYC, PTPN11*), while others are downregulated (*PTPN6, PIAS3* and *4, SPRED2*). Besides these steady genes, some JAK-STAT pathway related genes are variously regulated during ontogeny (*STATs, GRB2, CREBB*, etc.). *STAT1* and *STAT5A* gene expression is upregulated throughout ontogeny reaching maximum in erythroid progenitors of BM origin. *STAT5B* and *STAT6* gene expression is downregulated in most ontogenic stages reaching slightly positive values only in erythroid progenitors of BM- and FL-derived cells, respectively. The growth factor receptor-bound protein 2 (*GRB2*) demonstrates upregulation in erythroid cells of FL and BM origin compared to its downregulation in CB- and PB-derived cells. The enzymes phosphoinositide-3-kinase (*PIK3*), catalytic, gamma polypeptide (*PIK3CG*) and PIK3, regulatory subunit 2 (*PIK3R5*) are downregulated in all hematopoietic cells during ontogeny, whereas *PIK3R2* is downregulated only in BM-derived cells and upregulated in other hematopoietic cells. CREB binding protein (*CREBBP)* gene expression is absent in PB-, but has the highest elevation in FL- and CB-derived erythroid cells. BCL2-like 1 (*BCL2L1*) gene expression is downregulated in CB-derived cells and increased in other cells, mostly in PB-derived erythroid cells. *SOS1* gene expression is increased in CB- and BM-derived cells.

**Figure 4:**
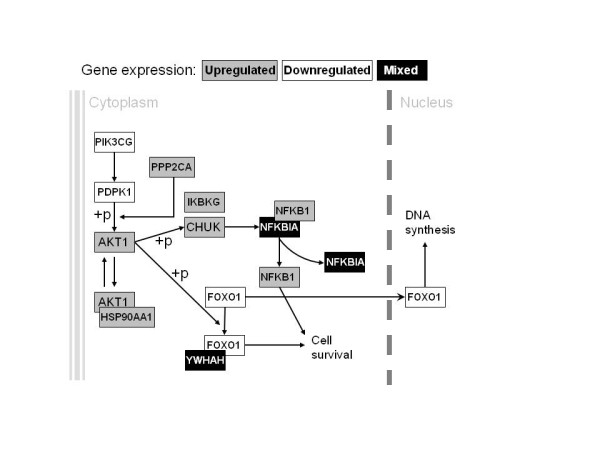
** AKT signaling pathway.** The expression of genes linked to AKT signaling pathway through ontogeny. (+p) phosphorylation, → stimulation, → translocation.

Phosphorylation of AKT, a signaling molecule downstream of PI3K, is observed following SCF treatment [16]. We use SCF in medium of our liquid cultures to induce erythroid differentiation of hematopoietic progenitors, together with EPO and cocktail of cytokines. We present PI3K-AKT signaling pathway related gene expression in Figure 4, as determined by microarray analysis. Beside continuously upregulated (*AKT1, PPP2CA, CHUK, NFKB1*, etc) and downregulated (*FOXO1, PDPK1, PIK3CG*) genes in the PI3K-AKT signaling pathway, we observe intermittently regulated gene expression (*YWHAH, NFKBIA*). Tyrosine 3-monooxygenase/tryptophan 5-monooxygenase activation protein (*YWHAH*) gene expression is absent in erythroid progenitors of FL origin, downregulated in CB- and BM-, and not changed in PB-derived cells. Nuclear factor of kappa light polypeptide gene enhancer in B-cells inhibitor, alpha (*NFKBIA*) gene expression is upregulated in erythroid progenitors of CB origin, downregulated in FL- and BM-, and not changed in PB-derived cells. *HSP90AA1* has the most increased expression of all PI3K-AKT pathway related genes, continuously increasing expression from fetal- to adult-derived erythroid cells. In contrast, upregulated *PPP2CA* gene expression demonstrates continuous decrease from fetal- to adult-derived erythroid cells.

## Discussion

We present the number of genes overexpressed in erythroid progenitor cells from several ontogenic stages. The total gene expression in erythroid progenitor cells of CB-derived tissues is twice that in other examined cells. These erythroid progenitor cells share 1011 common genes in cells derived from all examined ontogenic stages. We perform statistical analysis of the common genes among examined ontogenic stages and determine significant *HDAC1* and *SERPINB1* upregulation in FL-derived and significant *ERAF, STAT5A* upregulation in adult cells-derived erythroid progenitors. *ERAF* gene expression is directed by EPO and SCF activity, while *STAT5A* promote proliferation and growth of erythroid cells through JAK-STAT pathway. We also perform functional categorization by Ingenuity Pathways analysis with the network of genes linked to hematological system development. This analysis reveal the *KITLG, EPO, GATA1, PIM1* and *STAT3* genes as the most important interaction points for activity of related genes involved in hematological development.

We focus our presentation of microarray results on pathways linked to hematological system development, cellular growth and proliferation. Besides already mentioned meeting point genes (*GATA1, EPO*), we describe genes differentially expressed in some stages of ontogeny (*ERAF, PIM1*). The phosphorylation of GATA1 is important for EPO-induced maturation of fetal liver erythroid progenitor cells [23]. The role of GATA1 in terminal erythroid differentiation includes suppression of *GATA2* expression and upregulation of erythroid-specific target genes including those for *KLF1, NFE2* and *EPOR*. *GATA1* gene expression was low in our FL-derived erythroid cells, in contrast to other stages of ontogeny. ERAF is an erythroid-specific protein, with low levels in erythroid cells of FL-origin and high levels in BM-derived cells. In addition, HDAC1 and HDAC2 stimulation of cell proliferation is mediated by STAT3 [24], while HDACs gene expression is elevated in erythroid progenitors during ontogeny demonstrating importance in early stages of ontogeny. PIM family genes have a role in signal transduction in blood cells, contributing to both cell proliferation and survival. A recent report suggests that the PIM family genes, implicated in cytokine-dependent signaling in hematopoietic cells, are related to RUNX genes which regulate cell proliferation and differentiation in ontogeny [25]. *Runx1* is reported to play an early role in hematopoietic development, and we detect its increased gene expression in CB-derived erythroid cells [26].

According to previous reports, the PI3K-AKT signaling pathway regulates EPO-induced survival, proliferation, and maturation of early erythroid progenitors [27,28]. PI3K prevents apoptosis and stimulate cell proliferation in response to EPO stimulation in erythroid progenitors [29]. Both EPO and SCF induce activation of PI3K, moreover SCF causes activation of anti-apoptotic AKT, a signaling molecule downstream of PI3K [16,30]. In presented AKT signaling pathway, we demonstrate that CB-derived erythroid cell survival is related to upregulated *NFKBIA* gene, while FL-derived erythroid cell survival is linked to upregulated *NFKB1* and *PPP2CA* genes. Moreover, BM- and PB-derived erythroid cell survival is associated with elevated *HSP90AA1* gene expression.

Influence of FBS and cytokines on in vitro erythropoiesis has been observed in previous studies. It has been studied GM-CSF and IL-3 effects on adult human erythroid progenitors, stimulated to terminal differentiation by EPO, under FBS-supplemented or FBS-deprived culture conditions. Although hemoglobinization and maturation of BFU-E-derived erythroblasts was comparable in FBS-replete versus FBS-deprived cultures, the latter had significantly less γ-globin gene expression. Factors present in FBS appear to exert a dominant influence on fetal globin synthesis in vitro [31]. Both GM-CSF and IL-3 exhibit erythroid burst-promoting activity in FBS-deprived cultures, but IL-3 is more active [32]. Fetal calf serum (FCS) is known to elevate γ-globin mRNA levels and fetal hemoglobin in BFU-E culture; removal of FCS from the BFU-E cultures did not significantly reduce γ-globin mRNA levels [33]. FCS factors, responsible for fetal hemoglobin increase, act at both early and late stages of erythroid differentiation [34].

A recent report revealed that a persistent activation of transcription factor/signaling protein STAT5A in human hematopoietic stem and progenitor cells favored their erythroid differentiation [35]. Increased *STAT5A* gene expression is present throughout ontogeny and reached a peak in BM-derived erythroid cells. The tyrosine phosphorylation of STAT5, a downstream target for the non-receptor tyrosine kinase, JAK2, is mediated by EPO [16,17]. The *EPOR* gene has more than double expression in CB-derived erythroid cells in comparison to PB-derived cells according to our microarray analysis. *CREB1* is significantly elevated only in erythroid progenitor cells of FL origin, compared to other ontogenic stages. The protein tyrosine phosphatase family (PTP) contains signaling molecules that regulate cell growth and differentiation. *PTPN11* gene expression is elevated throughout ontogeny, reaching maximum in erythroid progenitors of BM origin. According to JAK-STAT pathway related gene expression in erythroid progenitors during different periods of ontogeny, we demonstrate the antiapoptotic mechanism regulated by *PIK3R2* (increased in fetal derived cells) and *BCL2L1* (increased in adult derived cells). The same apply for growth and proliferation where largely increased *PIM1, STAT1* and *STAT5A* gene expression is present throughout ontogeny and particularly in BM-derived erythroid progenitors. MAPK signaling pathway has been regulated by *PTPN11* (BM-derived), *GRB2* (FL- and BM-derived) and *SOS* (CB-derived cells) gene interaction.

## Conclusions

This ontogenic overview of specific genes and transcriptional programs in normal erythropoiesis may contribute to understanding of erythropoietic progenitor cell development. It may form the basis for modifications of gene expression in any kind of hematological malignancies as well as in other diseases affecting erythropoiesis, and more broadly, hematopoiesis. It will be important to extend this study of analyzed signaling pathways, from the gene expression level to protein expression and phosphorylation levels. Affirmation of examined genes on both levels will support the significance of our results.

## Competing interests

The authors declare that they have no competing interests.

## Authors’ contributions

VPC carried out experimental work described in the paper, participated in designing the study, drafted the manuscript and performed the statistical analysis. BB carried out the molecular genetic studies. BBC drafted the manuscript and performed the statistical analysis. CTN participated in designing the study and helped to draft the manuscript. RKP conceived of the study, and participated in its design and helped to draft the manuscript. ANS participated in designing the study and helped to draft the manuscript. All authors read and approved the final manuscript.

## Supplementary Material

Additional file 1 Statistically significant by t-test genes up-regulated vs. HuURNA among examined cells. Click here for file

Additional file 2 Statistically significant by t-test genes down-regulated vs. HuURNA among examined cells. Click here for file

Additional file 3 Statistically significant by t-test genes down-/up-regulated vs. HuURNA among examined cells. Click here for file

Additional file 4 Statistically significant genes by ANOVA down-/up-regulated vs. HuURNA among examined cells. p < 0.01 (shadow box), p < 0.05 (open box). Click here for file
