# Supplementary material for: JAK-STAT and AKT pathway-coupled genes in erythroid progenitor cells through ontogeny
Source: J Transl Med. 2012 Jun 7;10:116. doi: 10.1186/1479-5876-10-116 (PMC3412720; doi:10.1186/1479-5876-10-116)
Supplement: Additional file 1 — Statistically significant by t-test genes up-regulated vs. HuURNA among examined cells. [file 1479-5876-10-116-S1.doc]

**Supplemental table 1. Statistically significant genes up-regulated vs. HuURNA among examined cells.**

| **Gene Name** | **Gene Description** | **Same pattern as** |
| --- | --- | --- |
|
| ATP5L | ATP synt H^+^ transp mitoch F_0_ compl ngemp | HSPA5 |
| GGH | γ-glutamyl hydrolase (conjugase) | -//- |
| SF3B2 | splicing factor 3b, subunit 2, 145kDa | -//- |
| DENR | density-regulated protein | BAT2D1 |
| KDM3B | lysine (K)-specific demethylase 3B | -//- |
| SFRS5 | splicing factor, arginine/serine-rich 5 | -//- |
| TOMM20 | transl of outer mitoch membr 20 hom ngemp | -//- |
| YY1 | YY1 transcription factor | -//- |
| GAPDH | glyceraldehyde-3-phosphate dehydrogenase | ENO1 |
| **ENY2** | enhancer of yellow 2 homolog (Drosophila) | ERAF |
| **GSTO1** | glutathione S-transferase omega 1 | -//- |
| **HMGB2** | high-mobility group box 2 | -//- |
| HPS4 | Hermansky-Pudlak syndrome 4 | -//- |
| HSD17B10 | hydroxysteroid (17-beta) dehydrog10 ngemp | -//- |
| METTL13 | methyltransferase like 13 | -//- |
| Rbm17 | RNA binding motif protein 17 | -//- |
| **UCP2** | uncoupl prot 2 (mitoch proton carrier) ngemp | -//- |
| **RPS13** | ribosomal protein S13 | FADS2 |
| TAF15 | TAF15 RNA polymerase II, TATA box binding protein-associated factor, 68kDa | -//- |
| TTC3 | tetratricopeptide repeat domain 3 | NFATC3 |
| **SLC40A1** | solute carrier fam. 40 iron-regul transport m1 | MYBL2 |
| **TCEA1** | transcription elongation factor A (SII), 1 | -//- |

nuclear gene encoding mitochondrial protein (ngemp), member (m); Bolded genes – expression >1.5 fold vs. HuURNA; *increased significance to p<0.01;
