# Supplementary material for: JAK-STAT and AKT pathway-coupled genes in erythroid progenitor cells through ontogeny
Source: J Transl Med. 2012 Jun 7;10:116. doi: 10.1186/1479-5876-10-116 (PMC3412720; doi:10.1186/1479-5876-10-116)
Supplement: Additional file 2 — Statistically significant by t-test genes down-regulated vs. HuURNA among examined cells. [file 1479-5876-10-116-S2.doc]

**Supplemental table 2.** Statistically significant genes down-regulated vs. HuURNA among examined cells.

nuclear gene encoding mitochondrial protein (ngemp), member (m); Bolded genes – expression >1.5 fold vs. HuURNA; *increased significance to p<0.01;

| **Gene Name** | **Gene Description** | **Same pattern as** |
| --- | --- | --- |
| FCER2 | Fc fragm of IgE, low affin II, recept for CD23 | ADRB3 |
| PPFIA4 | protein tyrosine phosphatase, rec type, f polypept, α4 | DVL3 |
| BAZ1B | bromodomain adjacent to zinc fing. dom, 1B | CABP2 |
| EPS15L1 | Epider grow fact recept path substr 15-like1 | -//- |
| SLC37A1 | solute carrier fam 37, glyc-3-phosph transp | -//- |
| FAM173B | CDNA, FLJ79216 complete cds | BTRC |
| RDH11 | Vesicle soluble NSF attachment prot recept | -//- |
| UBE2CBP | Ubiquitin-conjugating enzym E2C bind. prot. | -//- |
| ERGIC1 | Endopl reticul-Golgi intermed compart prot1 | PDGFRA |
| RAPSN* | 43kDa acetylcholine receptor-associated proteine | -//- |
| **HLA-DRB1** | major histocompatib complex, class II, DRβ1 | F2R |
| PTPN6 | protein tyrosine phosphat, non-recept type 6 | -//- |
| FAM126B | family with sequence similarity 126 m B | GSTM1 |
| NKG7 | natural killer cell group 7 sequence | -//- |
| PLEKHO1 | pleckstrin homol domain contain, fam O m1 | -//- |
| PRKAR1A | prot kinase, cAMP-depend, regulat, type I, α | -//- |
| UCKL1 | uridine-cytidine kinase 1-like 1 | -//- |
| FAM129B | family with sequence similarity 129 m B | NPIPL3 |
| KRIT1 | Krit1 | ST3GAL1 |
| PCYT1B | phosphate cytidylyltransferase 1, choline, β | VAT1 |
| RNF24 | ring finger protein 24 | -//- |
