# Supplementary material for: JAK-STAT and AKT pathway-coupled genes in erythroid progenitor cells through ontogeny
Source: J Transl Med. 2012 Jun 7;10:116. doi: 10.1186/1479-5876-10-116 (PMC3412720; doi:10.1186/1479-5876-10-116)
Supplement: Additional file 3 — Statistically significant by t-test genes down-/up-regulated vs. HuURNA among examined cells. [file 1479-5876-10-116-S3.doc]

**Supplemental table 3.** Statistically significant genes down-/up-regulated vs. HuURNA among examined cells.

| **Gene Name** | **Gene Description** | **Same pattern as** |
| --- | --- | --- |
| GRB2 | growth factor receptor-bound protein 2 | CORO1A |
| GSTM4 | glutathione S-transferase mu 4 | -//- |
| COX17 | COX17 cytoch c oxidas assem hom, ngemp | PSMB6 |
| DULLARD | dullard homolog (Xenopus laevis) | -//- |
| UCRC | ubiquinol-cytochrome c reductase complex | -//- |

nuclear gene encoding mitochondrial protein (ngemp), member (m); *increased significance to p<0.01;
