# Supplementary material for: JAK-STAT and AKT pathway-coupled genes in erythroid progenitor cells through ontogeny
Source: J Transl Med. 2012 Jun 7;10:116. doi: 10.1186/1479-5876-10-116 (PMC3412720; doi:10.1186/1479-5876-10-116)
Supplement: Additional file 4 — Statistically significant genes by ANOVA down-/up-regulated vs. HuURNA among examined cells. p < 0.01 (shadow box), p < 0.05 (open box). [file 1479-5876-10-116-S4.doc]

**Supplemental table 4.** Statistically significant genes by ANOVA down-/up-regulated vs. HuURNA among examined cells. p<0.01 (shadow box), p<0.05 (open box).

| **Gene Name** | **Description vs.** | **F** | **F** | **F** | **C** | **C** | **B** |
| --- | --- | --- | --- | --- | --- | --- | --- |
|  |  | **C** | **B** | **P** | **B** | **P** | **P** |
| ATP5L | ATP synthase, H+ transporting, mitochondrial F0 complex, subunit G ngemp |  |  |  | > |  |  |
| BAT2D1 | BAT2 domain containing 1 |  |  |  |  |  | > |
| CDK2AP2 | cyclin-dependent kinase 2 associated protein 2 |  |  |  |  |  | < |
| CLEC4E | C-type lectin domain family 4, member E |  |  | > |  |  |  |
| **CORO1A** | coronin, actin binding protein, 1A |  |  | > |  |  |  |
| CORO1C | coronin, actin binding protein, 1C |  | < |  |  |  |  |
| DDOST | dolichyl-diphosphooligosaccharide-protein glycosyltransferase |  |  | > |  |  |  |
| ECH1 | enoyl Coenzyme A hydratase 1 peroxisomal |  |  | < |  |  |  |
| GADD45A | growth arrest and DNA-damage-inducible, α |  |  | < |  |  |  |
| HSD17B10 | hydroxysteroid 17-β dehydrogenase 10 ngemp |  |  | < |  |  |  |
| **IGFBP7** | insulin-like growth factor binding protein 7 |  |  | > |  |  |  |
| LDHA | lactate dehydrogenase A |  |  | > |  |  |  |
| NFATC3 | nuclear factor of activated T-cells, cytoplasmic, calcineurin-dependent 3 |  |  |  | < |  |  |
| PCYT1B | phosphate cytidylyltransferase 1, choline, β | > |  |  |  |  |  |
| PDLIM1 | PDZ and LIM domain 1 |  |  |  |  | > |  |
| PPA1 | pyrophosphatase (inorganic) 1 | < |  |  |  |  |  |
| RAPSN | 43kDa acetylcholine receptor-associated protein |  |  |  | < |  |  |
| RPN1 | ribophorin I |  |  | > |  |  |  |
| **SERPINB1** | serpin peptidase inhibitor, clade B member 1 |  |  | > |  |  |  |
| SLC40A1 | solute carrier family 40 (iron-regulated transporter)1 |  | < |  |  |  |  |
| SMAP2 | small ArfGAP2 |  | < |  |  |  |  |
| SMARCA2 | SWI/SNF related, matrix associated, actin dependent regulator of chromatin, subfamily a, m2 |  |  | > |  |  |  |
| ST3GAL1 | ST3 β-galactoside α-2,3-sialyltransferase 1 |  |  |  | < |  |  |
| STAT5A | Signal transducer and activator of transcription 5A |  | < |  |  |  |  |
| STAT6 | signal transducer and activ of transcr 6, IL-4 induc |  | > |  |  |  |  |
| **TIMP3** | TIMP metallopeptidase inhibitor 3 |  |  | > |  |  |  |
| TOMM20 | translocase of outer mitochondrial membrane 20 |  |  |  |  |  | > |
| **TPSB2** | tryptase beta 2 |  |  | < |  |  |  |
| TPST2 | tyrosylprotein sulfotransferase 2 |  | < |  |  |  |  |
| TUBG1 | tubulin, gamma 1 |  |  | < |  |  |  |
| UBXN1 | UBX domain protein 1 |  |  | < |  |  |  |
| VAT1 | vesicle amine transport protein 1 homolog |  |  | > |  |  |  |
| **VIM** | vimentin |  |  | > |  |  |  |
| WDR1 | WD repeat domain 1 |  |  | > |  |  |  |
| XBP1 | X-box binding protein 1 |  |  | > |  |  |  |
| YWHAZ | tyrosine 3-monooxygenase/tryptophan 5-mono-oxygenase activation protein, zeta polypeptide |  |  |  | < |  |  |

nuclear gene encoding mitochondrial protein (ngemp), member (m). Bolded genes – difference >1.5 fold. F-fetal liver, C-cord blood, B-bone marrow, P-peripheral blood.
